# An update on animal models of liver fibrosis

**DOI:** 10.3389/fmed.2023.1160053

**Published:** 2023-03-23

**Authors:** ShuTing Wu, XinXin Wang, WenBo Xing, FenYao Li, Ming Liang, KeShen Li, Yan He, JianMing Wang

**Affiliations:** ^1^Institute of Regenerative and Translational Medicine, Tianyou Hospital, Wuhan University of Science and Technology, Wuhan, China; ^2^Department of Hepatobiliary and Pancreatic Surgery, Tianyou Hospital, Wuhan University of Science and Technology, Wuhan, China

**Keywords:** liver fibrosis, liver injury, inflammation, *in vivo*, animal models

## Abstract

The development of liver fibrosis primarily determines quality of life as well as prognosis. Animal models are often used to model and understand the underlying mechanisms of human disease. Although organoids can be used to simulate organ development and disease, the technology still faces significant challenges. Therefore animal models are still irreplaceable at this stage. Currently, *in vivo* models of liver fibrosis can be classified into five categories based on etiology: chemical, dietary, surgical, transgenic, and immune. There is a wide variety of animal models of liver fibrosis with varying efficacy, which have different implications for proper understanding of the disease and effective screening of therapeutic agents. There is no high-quality literature recommending the most appropriate animal models. In this paper, we will describe the progress of commonly used animal models of liver fibrosis in terms of their development mechanisms, applications, advantages and disadvantages, and recommend appropriate animal models for different research purposes.

## 1. Introduction

For a long time, advances in biomedical research have often relied on the use of animal models as the basis for experimental and clinical hypotheses. The occurrence and development of various human diseases are very complex, and it is impossible and not allowed to conduct experimental research on human body to explore the pathogenesis, prevention and treatment mechanism of diseases. Therefore, animal models are frequently used to simulate and understand the underlying mechanisms of human disease. Organ fibrosis is the characteristic of the progression of chronic inflammatory diseases, which account for 45% of global all-cause mortality (1). Equally, the development of fibrosis primarily determines quality of life and prognosis in the liver (2). Liver fibrosis animal models are indispensable tools for studying the pathogenesis of liver fibrosis and developing therapeutic drugs. Although organoids can be used to simulate organ development and disease, they have wide applications in basic research, drug development and regenerative medicine (3–5). However, obtaining freshly isolated human hepatocytes is very limited and maintaining cultures in spinner flasks can be cost prohibitive, and hepatocyte maturation, culture longevity, and large-scale production of pure cultures remain challenges (6). Therefore, animal models are still irreplaceable at this stage.

**Figure 1:**
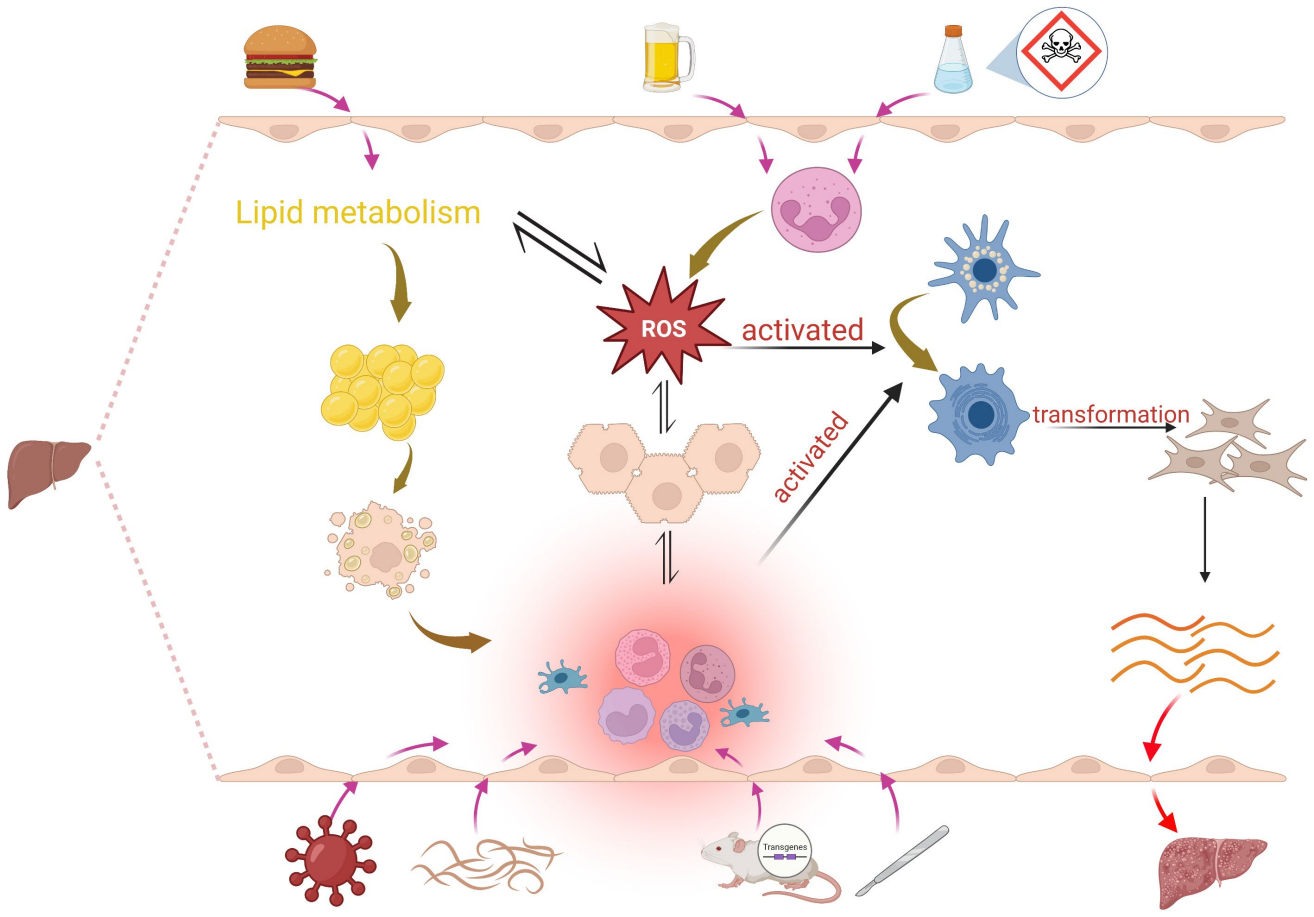
Mechanisms of induction in the liver fibrosis model: Chemical induction mainly leads to massive infiltration of neutrophils to produce ROS, which induces hepatocyte injury and activation of HSCs, and also interferes with lipid metabolism; hepatocyte injury significantly increases the production of immune cells such as cytokines, chemokine, Kupffer cells, B cells, T cells and ROS, and this pro-inflammatory environment and ROS activates HSC, promotes the conversion of HSC into myofibroblasts, and increased production of ECM proteins, which subsequently leads to liver fibrosis. Dietary induction mainly affects lipid metabolism, which leads to ROS as well as fat accumulation, and excessive accumulation of lipids in hepatocytes also activates inflammation. Surgery, transgenic, and immune induction induce liver fibrosis mainly by causing inflammation.

At present, there are five types of *in vivo* models of liver fibrosis: chemical, dietary, surgical, and transgenic and immune (7) (Figure 1). Animals commonly used to prepare models are mainly mice (8), rats (9), rabbits (10), Ossabaw pigs (11), macaques (12) and zebrafish (13). There are various animal models of liver fibrosis with different efficacy, which have different effects on the correct understanding of the disease and the effective screening of therapeutic drugs. There is currently no high-quality literature recommending the most appropriate animal model. This article will describe the research progress of commonly used animal models of liver fibrosis from the aspects of the development mechanism, application, advantages and disadvantages of animal models (Table 1) and recommend suitable animal models for different research purposes.

**Table 1:** Induction method, modeling time, and liver fibrosis in animal models of liver fibrosis.

Model	Induction method	Species	Method	Periodicity (weeks)	Liver injury	Inflammation	Fibrosis
**Chemical**							
Ethanol	Ethanol	rat/mouse	i.g.	8 ~ 70	Y	↑	↑
CCl_4_	CCl_4_	rat/mouse	i.p.	4 ~ 6	Y	↑↑	↑↑
TAA	TAA	rat	i.p.	12 ~ 13	Y	↑↑	↑↑
		macaque	s.c.	8	Y	↑↑	↑↑
		marmoset	s.c.	11	Y	↑↑	↑↑
Nitrosamines	DMN	rat	i.p.	4	Y	↑↑	↑↑↑
	DEN	rat/mouse	i.p.	4 ~ 6	Y	↑↑	↑↑↑
**Diet**							
MCD	MCD	mouse	p.o.	6 ~ 8	Y	↑↑	↑
HFD	HFD	mouse	p.o.	24 ~ 25	Y	↑	↑
	WD	Ossabaw pig	p.o.	16	Y	↑↑	↑
	FFD	mouse	p.o.	30	Y	↑↑	↑↑
CDAA	CDAA	rat/mouse	p.o.	12	Y	↑↑	↑
CDAHFD	CDAHFD	rat/mouse	p.o.	6 ~ 9	Y	↑↑	↑↑
**Surgical**							
BDL	BDL	rat/mouse	p.o.	4 ~ 5	Y	↑↑	↑↑
**Transgenic**							
Transgenic	Gnmt-	mouse	knockout	12	Y	↑↑	↑↑
Transgenic	Mdr2^−/−^	mouse	knockout	8 ~ 14	Y	↑↑	↑↑
**Immunity**							
Schistosoma	Schistosoma j	mouse	s.c.	8	Y	↑↑	↑↑
Virus	HBV	mouse	i.v.		Y	↑↑↑	↑↑
PS	PS	rat	i.p.	16 ~ 24	Y	↑↑	↑
Con A	Con A	mouse	i.v.	4 ~ 8	Y	↑↑	↑
**Composite**							
Chemical +Chemical	CCl_4_ + Ethanol	mouse	i.g. + i.p.	7	Y	↑↑↑	↑↑
Chemical +Diet	STAM	mouse	s.c. + p.o.	3 ~ 8	Y	↑↑	↑↑↑
	HFD + Ethanol	mouse	i.g. + p.o.	12	Y	↑↑	↑↑
	CCl_4_ + WD	mouse	i.p. + p.o.	12	Y	↑↑	↑↑↑
	TAA + FFD	mouse	i.p. + p.o.	8	Y	↑↑	↑↑↑
Transgenic +Diet	*ob/ob* + HFD	mouse	p.o.	20	Y	↑	↑↑
	adropin-KO + MCD	mouse	p.o.	4	Y	↑↑↑	↑↑
	adropin-KO + WD	mouse	p.o.	16	Y	↑↑↑	↑↑

“i.g.” represents intragastric administration; “i.p.” represents intraperitoneal injection; “s.c.” represents subcutaneous injection; “p.o.” represents per os; “i.v.” represents intravenous injection. “Y” means yes, the “↑.” indicates mild, “↑↑” indicates moderate, and “↑↑↑” indicates severe.

## 2. Chemical induction methods

The chemical injury liver fibrosis model is used to induce the formation of liver fibrosis by using chemical drugs to enter hepatocytes to produce toxic metabolites that cause persistent liver injury. Currently, this model preparation method mainly uses ethanol, carbon tetrachloride (CCl_4_), thioacetamide (TAA), dimethylnitrosamine (DMN), diethylnitrosamine (DEN) or other liver toxins to induce liver fibrosis models.

### 2.1. Alcohol-induced liver fibrosis model

The liver is the main organ involved in alcohol metabolism. Fibrosis associated with alcoholic liver disease is caused by multiple mechanisms, including acetaldehyde accumulation, reactive oxygen species (ROS) and hepatic overload of endogenous lipopolysaccharide (LPS) (14). Related research has shown that chronic alcohol abuse leas to overproduce of ROS and interferes with lipid metabolism in the liver, resulting in ROS-mediated liver injury (15). It is supposed that fibrosis is promoted by neutrophils through ROS production inducing hepatocyte injury and hepatic stellate cells (HSC) activation (16). Moreover, alcohol-stimulated liver fibrosis is the result of strong immune response involving many types of hepatocyte and different signal transduction pathways (17). Alcohol-induced liver injury significantly increases the production of cytokines, chemokines, other soluble mediators and components of the innate immune system, this pro-inflammatory environment leads to the activation of HSC and myofibroblast, increases the production of extracellular matrix (ECM) proteins, which can subsequently induce fibrosis in the liver (18).

Alcohol *ad libitum* feeding model is one of the earliest animal models used for alcoholic liver disease research in rodents (19). The concentration of ethanol solution is at 10–40% (v/v), and the alcohol administration cycle used in different groups is from 8 weeks to 70 weeks, there is no significant change in mortality (20). In most studies, models of alcohol *ad libitum* feeding can sufficiently induce liver injury, and accompanied by significant steatosis and exaltation in aspartate aminotransferase and alanine aminotransferase, but no more advanced fibrotic or cirrhotic lesions (20, 21). Because mice are naturally adverse alcohol; methods of feeding alcohol-containing liquid food are greatly limited. At present, the more commonly used method is alcohol combined with chemical gavage, which replicates the alcoholic liver fibrosis model while controlling the diet, this model has the advantages of simple operation, short cycle, high forming rate (22).

### 2.2. CCl_4_-induced liver fibrosis model

CCl_4_ has been widely used to induce mice liver injury and fibrosis for decades (23). High dose (≥1 ml/kg) of CCl_4_ can lead to reproducible acute liver injury. Toxicity of CCl_4_ is dependent on the P450-catalyzed metabolism to the reactive metabolite trichloromethyl radical (CCl_3_), and CCl_4_ is converted into ·CCl_3_ to bind to proteins, deoxyribonucleic acid (DNA) and lipids, which can cause mitochondrial damage and oxidative stress. ·CCl_3_ can also react with O_2_ to form trichloromethylperoxy radical (CCl_3_OO), thereby initiating lipid peroxidation chain reaction and destroying cell membrane (24).

Liver fibrosis is induced by intraperitoneal injection of CCl_4_ administered 2–3 times a week for 4–6 weeks in most research protocols. Bubnov et al. (9) injected freshly prepared 50% CCl_4_ hydrated olive oil solution into the rat intraperitoneally. On the 8th week after injection observed ultrasound manifestation of advanced liver fibrosis, including hepatosplenomegaly, portal hypertension, demonstrating that Carbohydrate tetrachloride induces injury of liver parenchyma evoking fast and severe liver fibrosis. CCl_4_ treatment increased serum aspartate aminotransferase and alanine aminotransferase levels, produced hepatic oxidative and nitrative stress, and evoked profound expression of pro-inflammatory cytokine expressions in liver tissue (25). Moreover, the animals of CCl_4_ treatment exhibited higher apoptosis and showed obvious fibrosis in animal liver (25). Research showed that the non-specific liver inflammation triggered by CCl_4_ recruited high numbers of CD4^+^ T, CD8^+^ T and B cells, and elevated the expression of proinflammaitory cytokines in mice, further breaking liver tolerance and inducing autoimmune response, Autoimmune hepatitis inflammation and liver fibrosis in the presence of CYP2D6 antigen mimicry (26).

The advantages of the CCl4-induced fibrosis model are the relatively low cost of development, the relatively simple method of implementation, the short duration of induction, and the significant pathological changes in the liver tissue, which can be reversed even after cessation of drug administration (23). This model is a representative and reproducible model of liver fibrosis and is frequently used in the research of liver fibrosis development and the research of liver repair mechanism. However, the disadvantage of this model is that the animals cannot become obese or develop insulin resistance (IR), which is different with pathophysiological features of non-alcoholic fatty liver disease (NAFLD) patients induced by metabolic disorder (27). Furthermore, CCl_4_ is highly toxic and volatile, requiring researchers to take appropriate safety measures.

### 2.3. TAA-induced liver fibrosis model

TAA is a classic liver toxin and also a potent carcinogen and mutagen, which can induce oxidative stress and sterile inflammation, leading to acute and chronic liver injury (28, 29). TAA induces hepatotoxicity in mice and rats at doses ≥100 mg/kg. It is converted to metabolites TAA S-oxide and S, S-dioxide by cytochrome P450 enzymes and S, S-dioxide initiates toxicity by binding to lipids and proteins (24). TAA-induced liver injury is mainly caused by reaction metabolites secreted by TAA, which not only activate HSC, but also produce fibrinogen and growth factors, aiming to promote acute liver injury and chronic liver fibrosis (29).

TAA-induced liver fibrosis is a widely used model, and TAA can be administered orally or by intraperitoneal injection. But intraperitoneal injection provides more consistent results. Many researches have used Sprague–Dawley (SD) rats to induce liver fibrosis by intraperitoneal injection of TAA at a dose of 200 mg/kg twice a week for 12–13 weeks (30, 31). Matsuo et al. (12) used healthy macaca fascicularis to induce fibrosis model, dissolving TAA in normal saline and administrated three times a week at a dose of 100 mg/kg, and obtained that the TAA-induced model was superior to the CCl_4_ model. It both induced liver fibrosis progression and worsened residual liver function, but there were also individual differences in the effect of the reagent and the inability to assess whether reversal of fibrosis would occur after cessation of the reagent. Inoue et al. (32) have developed a marmoset hepatic fibrosis model for regenerative medicine research. The female marmosets were administered TAA at a dose of 2.5–40 mg/kg two or three times a week, lasting 11 weeks, the results suggest that continuous TAA administration induces persistent hepatic fibrosis in the common marmoset and this nonhuman primate hepatic fibrosis model have the possibility to evaluate the therapeutic effects of test samples to ameliorate hepatic fibrosis.

TAA-induced liver fibrosis is very similar to human liver fibrosis in terms of hemodynamic, morphological and biochemical metabolism (33). TAA disrupts DNA, RNA and protein synthesizing enzymes in hepatocytes, leading to metabolic disturbances and hepatocyte necrosis, a distinctive feature of this model compared to the CCl4 model, whose fibrosis remains stable for several weeks after TAA withdrawal. However, TAA is a carcinogen, which is both toxic and volatile (34).

### 2.4. Nitrosamines-induced liver fibrosis model

#### 2.4.1. DMN-induced liver fibrosis model

DMN is a powerful liver toxin, which can lead to liver injury, and provides a suitable experimental rat modeling reagent for liver fibrosis. The metabolic activation and detoxification of DMN cause hepatocyte injury, inflammation, neutrophil infiltration, and massive hepatic necrosis, which results in oxidative stress and production of ROS. These processes induce activation of hepatic stellate cells and increased synthesis of connective tissue components, especially collagens that end up in hepatic fibrosis (35). DMN not only induces liver fibrosis, but also can lead to cirrhosis due to repeated exposure to low doses in animals (36).

Many studies induced fibrosis in male SD rats by intraperitoneal injection of DMN at a dose of 1 ml per 100 g body weight per week, 3 days per week for 4 weeks (37–39). Repeated exposure to low doses of DMN results in subacute or chronic liver injury with varying degrees of necrosis, fibrosis, and nodular regeneration (40). DMN can cause acute liver injury in rats and reproduce the characteristics of human liver fibrosis and cirrhosis, as well as collagen accumulation, hepatocyte apoptosis, elevated oxidative stress and lipid peroxidation (41).

DMN-induced liver fibrosis rat model is a commonly used animal model to study liver injury diseases. Due to its short modeling time and low mortality, the formation of liver fibrosis is stable and is very similar to the characteristics of early changes and collagen fibrosis deposition of human liver fibrosis, and it is not easy to spontaneously resolve and recover after the cessation of exposure, so it is one of the classic animal models for studying the pathological mechanism, serum marker evaluation and drug therapy of liver fibrosis (42). However, researchers should ensure appropriate safety measures are in place due to the toxicity of nitrosamines.

#### 2.4.2. DEN-induced liver fibrosis model

DEN is considered to one of the most toxic drugs, which can result in various forms of necrosis and subsequent fibrosis (43). DEN has been shown to induce severe liver injury by inducing mutant DNA damage and upregulating ROS production (44). Furthermore, DEN administration results in excessive deposition of ECM protein (collagen) in rat liver and seems appropriate to study early events associated with the development of liver fibrosis (45). Some studies induced liver fibrosis by intraperitoneal injection of DEN in rats once a week for 4–6 weeks (46, 47).

DEN, a known carcinogen that leads to significant oxidative stress and DNA mutations, enhances lipotoxicity and accelerates the progression of fibrosis and cirrhosis, has long been used in hepatocellular carcinoma (HCC) models (48). Chen et al. (49) studied a DEN-induced cirrhosis mouse model, in which male C57BL/6 mice were given 0.014% DEN in drinking water 6 days a week, 1 day interval from normal drinking water, for 15 weeks. In this model, all mice given oral DEN developed liver fibrosis, cirrhosis, and HCC, and the histological pattern in the model was similar to that described in humans. DEN-induced rat HCC, which presents a stepwise histopathological progression similar to human HCC, was used to analyze different stages of inflammation, fibrosis, and cancer. Ding et al. (50) injected DEN in rats at a dose of 30 mg/kg body weight twice a week for 11 weeks and the animals were observed until week 20. The results suggested that the model characterized resulted in three stages: the inflammation stage (week 2–6), the fibrosis stage (week 8–12), and the HCC stage (week 14–20).

### 2.5. Other liver toxins

Other liver toxins such as arsenic (As), acetaminophen (APAP), and d-galactosamine (D-GalN) can also induce liver fibrosis.

As is an environmental toxicant and human carcinogen, and the liver is the main target organ for arsenic toxicity. As and its metabolites are toxic to hepatocytes, causing DNA damage and generating several free radicals. Free radicals subsequently induce lipid peroxidation, which may lead to cellular dysfunction or directly attack cells, triggering their damage (51). Repeated damage and repair of hepatocytes leads to liver fibrosis (52). As exposure causes liver injury in rats and liver fibrosis increases with increasing dose and time (53). Arsenite-induced liver fibrosis is a slow disease process in which many cellular and inflammatory factors are involved, including hepatocyte water degeneration, hepatocyte balloon formation, hepatocyte necrosis (inflammatory infiltration), hepatocyte regeneration, fibrous tissue proliferation, and liver fibrosis (53). Arsenite exposure induced HSC activation and extracellular matrix deposition, and long-term exposure to arsenite induced liver damage, inflammation, and fibrosis in mice or rats (54–56). Wang et al. (52) fed SD rats at a high dose of 100 mg/kg and exposed to sodium arsenite, cell swelling, inflammatory cell infiltration, and fibrous proliferation were evident.

APAP is a major cause of hepatic failure (57). The vast majority of ingested APAP is glucuronidated or sulfated and rapidly excreted. However, a small fraction is metabolized by hepatic cytochrome P450 enzymes to the highly reactive intermediate N-acetyl-p-benzoquinone mine (NAPQI), which is usually detoxified by glutathione (GSH)-coupled detoxification. In the initial stages of APAP liver injury, NAPQI depletes GSH stores and adds sulfhydryl adducts to cellular proteins (58). The resulting oxidative stress, mitochondrial uncoupling, adenosine triphosphate (ATP) depletion and c-Jun N-terminal (JNK) activation eventually lead to hepatocyte necrosis (58, 59). Related studies have shown that administration of repeated doses of APAP induces liver fibrosis (60, 61).

Acute co-injection of LPS/D-GalN is a widely used experimental model for acute liver injury, while long-term and low-dose treatment with LPS/D-GalN induces a chronic inflammatory response similar to that of liver fibrosis (62). Liver injury caused by a large depletion of uracil nucleotides, resulting in reduced RNA and protein synthesis, is mostly used to induce acute liver injury with a high degree of fibrosis, mostly in stages III to IV, with high similarity to human liver fibrosis and good reproducibility, but the disadvantage is the high time and cost consumed by modeling (63).

## 3. Diet induction methods

Many diseases are influenced by dietary factors, and simulating daily meals helps prepare animal models that are more closely related to the clinical manifestations of human diseases. The model preparation methods mainly include methionine choline-deficient diet (MCD), high-fat diet (HFD), Western diet (WD), choline-deficient, l-amino acid-defined (CDAA), and choline-deficient, l-amino acid-defined, high-fat diet (CDAHFD).

### 3.1. MCD-induced liver fibrosis model

A standard MCD contains 40% high sucrose and 10–20% fat. The deficiency of two essential nutrient, choline and methionine, lead to impaired fatty acid β oxidation and impaired production of very low density lipoprotein particles (64). In addition, choline deficiency leads to impaired hepatic very low density lipoprotein secretion, resulting in hepatic fat accumulation, hepatocyte death, oxidative stress, and changes in cytokines and adipokines, but causes only slight hepatic inflammation and fibrosis (65). After addition of methionine deficiency, there will be more pronounced inflammation and early development of fibrosis (after 8–10 weeks) (64).

Dietary animal models are widely used to research nonalcoholic steatohepatitis (NASH) pathogenesis, and mice fed the MCD diet are the preferred method (66). Feeding mice with the MCD diet is a mature nutritional model of NASH, which elevates serum transaminases, and liver histological changes similar to human NASH, including hepatic steatosis, lobular inflammation, and pericellular fibrosis (67). This model provides Histological marker of NASH because it is prone to transition from simple steatosis to steatohepatitis and can reach fibrosis stages (68). Many studies induce NASH (69, 70) and dietary liver fibrosis (71) by feeding mice MCD diet for 6–8 weeks. The gene expression of inflammatory markers in the MCD diet animal model occurs much earlier than that in the HFD animal model and can spontaneously develop liver injury characterized by fibrosis patterns within a short period of time (72). Furthermore, the MCD diet is able to induce significant changes in the expression of genes that encode proteins involved in the fibrogenesis pathway much earlier than HFD and most of the related genes, such as COL1A1, COL1A2, MMP-9, MMP-13, TIMP-1, and TGF-β, were upregulated within 2 weeks of feeding with the MCD diet (72).

The advantage of MCD dietary model is that it is more efficient and reproducible for inducing severe liver injury and progressive fibrosis; this dietary approach, which mimics a subgroup of NASH patients with advanced histological NASH, is ideal for studying the mechanisms driving NASH-associated inflammation/fibrosis and strategies for inhibiting these processes (71) and can be used to screen drugs that directly target liver fibrosis (73), and it is widely available. Moreover, steatohepatitis and fibrosis was induced in a shorter time (less than 10 weeks) than HFD model, increased pro-inflammatory cytokine levels and oxidative stress (74). But the MCD diet also has certain drawbacks, as it leads to weight loss and does not induce characteristics of metabolic syndrome, which is an important risk factor for NAFLD (75). Although a non-physiological diet low or deficient in certain essential amino acids promotes more severe fibrosis, it also leads to significant weight loss, making these NASH models more suitable for detecting the effects of drug therapy on liver injury and regeneration (76).

### 3.2. High-fat diet-induced liver fibrosis model

#### 3.2.1. HFD-induced liver fibrosis model

Many diet-induced obesity models mimic the natural history of NASH and show relatively good clinical translatability in terms of key metabolic and hepatic pathological changes in mild to moderate liver fibrosis, so these models are increasingly used in preclinical drug development (76). The use of high fat content alone is often referred to as the HFD model (77). Animal HFD usually include 45% energy-supplying high-fat diets and 60% energy-supplying high-fat diets. A HFD enhances glycolysis and accelerates NAFLD fibrosis progression by downregulating geranylgeranyl diphosphate synthase (GGPPS) expression; chronic HFD overload decreases GGPPS expression in mice, thereby shifting fuel preference from fatty acids to glucose; liver-specific GGPPS deficiency drives the Warburg effect by impairing mitochondrial function, which then induces liver inflammation, thereby exacerbating fibrosis (78). Transcription and protein levels of IL-1 were significantly increased in the liver of HFD-fed mice, and excessive accumulation of lipids in hepatocytes activates inflammation. The inflammatory process leads to an increased level of TGFβ and activation of β-catenin signaling pathways promoting epithelial-mesenchymal transition, which leads to acquisition of mesenchymal features and induces hepatic fibrosis (79).

Most studies about animals fed an HFD diet for less than 4 months showed that no significant changes in gene expression of proteins involved in fibrogenesis pathway, but it found that these have significant changes in studies with longer HFD exposure (24–25 weeks) (80, 81). HFD animal models require prolonged feeding with HFD to stimulate the progression of steatosis to mild steatohepatitis (72). Although long-term HFD feeding caused obesity and IR in mice, two key risk factors of NASH (82), it only mimicked the gene expression profile and histopathology of simple steatosis, not stimulated the gene expression profile and histopathology of NASH (83, 84). HFD-fed animal models can mimic metabolic abnormalities of NAFLD, other spectrums of oxidative stress and inflammation, but fail to reach advanced stages, such as fibrosis and cirrhosis (68). It is well known that only HFD diet feeding mice will cause a lot of steatosis, but little liver fibrosis.

#### 3.2.2. WD-induced liver fibrosis model

A recent mouse model combining long-term administration of a “Western diet” with high saturated fat and cholesterol content was able to replicate NASH with increased but not inflated fibrosis markers (85). The WD is a diet rich in saturated fats, trans fats and table sugar (86) and represents a cholesterol-added HFD that mimics the fast-food diet (FFD) associated with the pathogenesis of NASH in humans (80).

Panasevich et al. (11) fed juvenile female Ossabaw pigs with WD and developed severe NASH after 16 weeks with hepatic steatosis, hepatocyte ballooning, inflammatory cell infiltration and fibrosis, histological inflammation and fibrosis after 36 weeks of WD feeding further deteriorated. The WD model mimics the vast majority of obese NAFLD/NASH patients who typically have IR and metabolic syndrome but relatively mild liver damage. Therefore, the WD model should be the first choice for studying how NAFLD/NASH affects systemic metabolic and cardiovascular risk of tissue complications with type 2 diabetes and atherosclerosis (71). Diet induced obese mice fed with WD are attractive because they summarize the natural history of NASH, and traditional obesogenic HFD promotes dyslipidemia, fatty liver, and mild NASH in rodents without significant fibrosis (87).

The lack of high levels of fructose in the Western diet may be physiologically important because adding high fructose content to a diet high in saturated fat and cholesterol has been thought to reproduce all the characteristics of NASH. Tsuchida et al. (8) developed a new rodent model of NASH fibrosis based on a “fast food” (high cholesterol, high saturated fat and high fructose) diet administered for 6 months, outlining the characteristics of metabolic syndrome and NASH with progressive fibrosis in C57BL/6 mice. After Xin et al. (88) gave mice a high-fat, high-carbohydrate diet for 30 weeks, mice exhibited significant hepatic fibrosis, hepatic steatosis, ballooning degeneration and inflammation. Feeding C57BL/6 J mice a high-fat, high sucrose, high-cholesterol diet has been shown to induce features of human liver fibrosis such as steatohepatitis, hepatocyte ballooning, and progressive fibrosis (80). However, a major challenge in high-fat, carbohydrate diet models is the long dieting period (usually >20 weeks) required for the progression of steatohepatitis disease to hepatic fibrosis.

### 3.3. Choline-deficient L-amino-defined diet-induced liver fibrosis model

#### 3.3.1. CDAA-induced liver fibrosis model

Another formulation of the MCD diet is a CDAA diet. Like the MCD diet, the CDAA diet induced hepatic triglycerides accumulation by inhibiting the liver output of very low density lipoprotein and impairing fatty acid oxidation in hepatocytes and these inhibitory effects on lipid disposal are sufficient to increase lipid synthesis and oxidation and endoplasmic reticulum stress to stimulate hepatitis cell infiltration and HSC activation (89).

Related studies showed that C57BL / 6 J mice fed with CDAA diet gained the same or more weight than mice on a standard diet (90). The CDAA diet induces changes similar to human NASH in rats, such as steatohepatitis, fibrosis, cirrhosis and HCC, but has minimal effects on body weight and glucose metabolism compared to semi-purified MCD diet (91). CDAA diet-fed rats lack obesity and IR (92), and CDAA diet-fed mice exhibited obesity and IR develops limited liver fibrosis (93). Exogenous LPS administration exacerbates pericellular fibrosis in CDAA-mediate steatohepatitis in mice. Nakanishi et al. (94) fed C57BL/6 J mice a CDAA diet to induce NASH and intraperitoneally injected low-dose LPS (0.5 mg/kg) three times a week, LPS challenge potentiated CDAA-diet-mediated insulin resistance, hepatic steatosis with upregulation of lipogenic genes, and F4/80-positive macrophage infiltration with increased proinflammatory cytokines. LPS administration extensively promoted HSC activation in mice fed on a CDAA diet, thereby promoting pericellular fibrosis. Tølbøl et al. (95) have described a new rat NASH model of cholesterol-supplemented CDAA diet with severe fibrosis, which reflected the human NASH phenotype and disease progression, and stably induced the phenotype in short period of time. The CDAA diet had resulted in significant hepatomegaly and fibrosis after 4 weeks of feeding, with further development of collagen deposition and fibrogenesis-related gene expression during 12 weeks of feeding. Cholesterol supplements enhanced the stimulating effect of the CDAA diet on transcripts of genes associated with fibrogenesis without significantly increasing collagen deposition.

#### 3.3.2. CDAHFD-induced liver fibrosis model

CDAHFD is composed of 60 kcal % fat and 0.1% methionine by weight (96). Mice are largely resistant to the CDAA diet (97), but Chiba et al. (98) recently developed a modified CDAA diet that effectively induced NASH in mice by adding lard to reduce methionine and increase fat mass. Mice fed a 60% fat CDAA diet exhibited steatohepatitis with dietary fat-driven dysregulation of lipid metabolism-related genes, progressive fibrosis, and HCC (89).

Some study protocols induced rapid liver fibrosis development of NASH by feeding C57BL/6 mice with a CDAHFD diet for 12–15 weeks (99–101). After feeding a CDAHFD diet, mice showed higher serum alanine aminotransferase, aspartate aminotransferase and alkaline phosphatase levels, significantly increased serum CK18 levels, and also enhanced the pathological features of steatohepatitis and liver fibrosis (101). This model can rapidly and consistently develop liver fibrosis, steatosis and inflammation. Zhou et al. (102) fed adult male Wistar rats a CDAHFD diet for 9 weeks, and the model successfully induced fibrosis and steatosis in the rat liver. It has been reported that CDAHFD dietary models developed steatosis, steatohepatitis and liver fibrosis faster and more severe than traditional models and prevent weight loss in mice, but CDAHFD dietary models do not develop obesity (96).

## 4. Surgical induction methods

Bile duct ligation (BDL) is the most widely used and longest used experimental model for cholestasis because of its high reproducibility. This technique requires a mid-abdominal laparotomy and isolation of the common bile duct above the duodenum, followed by double ligation and dissection of the bile duct to produce a model of obstructive cholestasis (103). It induces proliferation of intrahepatic biliary epithelial cells, proliferation of myofibroblast differentiation of portal vein fibroblasts surrounding biliary epithelial cells, resulting in high reproducibility, high expression and deposition of ECM (104, 105). The BDL model shows liver injury manifested by histological changes and elevations in serum biochemistry, ductal reactions, fibrosis, and inflammation, leading to activation of Kupffer cells and recruitment of immune cells, possibly triggering an inflammatory response through activation of the NF-κB pathway (106).

Common BDL in rats or mice is a classic method to produce an animal model of liver fibrosis (107). The application of this model in rats and mice is popular among scientists who aim to understand the pathogenesis of liver inflammation and fibrosis. BDL in mice is a model widely used to induce biliary inflammation, fibrosis and cholestatic liver injury (108). Meier et al. (109) anesthetized male DBA-1 mice with isoflurane, performed midline laparotomy, dissected common bile duct and cut between four ligations under anatomical microscope to induce fibrosis in mice. It was reported that sinusoidal and portal fibrosis had fully developed on days 10 and 20 after BDL surgery in mice, respectively (103). Significant bile duct proliferation and dilated portal fibrosis were observed in all mice included in the study 5 weeks after BDL surgery in mice (110). Matyas et al. (111) demonstrated that BDL-induced advanced liver fibrosis is a suitable mouse model to study the pathophysiology of cirrhosis and cardiomyopathy at the preclinical level, as it resembles the characteristics of the clinical syndrome in patients. BDL induced massive inflammation, oxidative stress, microvascular dysfunction, and fibrosis in the liver, and these pathological changes were accompanied by impaired diastolic, systolic, and macrovascular functions, cardiac inflammation, and oxidative stress. Schewe et al. (112) induced liver fibrosis in male SD rats by BDL surgery for 4 weeks. After BDL surgery, the liver showed low fibrosis and severe bile duct proliferation, accompanied by overall parenchymal fibrosis and moderately inflammatory fibrous septum. These modifications were typical features of BDL and were characteristic of liver fibrosis (113).

The BDL model is mainly used to evaluate the study of cholangiocyte proliferation, apoptosis and portal fibrosis due to extrahepatic cholestasis (114). Because fibrogenesis and liver regeneration proceed simultaneously in the BDL model (115), this model is also an ideal tool to evaluate the protective effect of liver regeneration on fibrosis. Marques et al. (113) suggested that BDL was considered a safer method to induce cirrhosis in rats compared with the use of CCl_4_, inducing cirrhosis after 4–6 weeks. However, mortality due to bile leakage and gallbladder (or mouse gallbladder) rupture that may occur during BDL is relatively high (11), and BDL is much more painful than CCl_4_-induced liver injury (116).

## 5. Transgenic induction methods

A number of transgenic animal models have been developed for the study of liver fibrosis based on the different pathogenesis of liver fibrosis and the key functional genes regulated by liver fibrosis. Sterol regulatory element-binding protein-1c transgenic mice developed severe IR and NASH, with perivenular and pericellular fibrosis, but reduced adipose tissue volume (64). Gnmt-deficient (Gnmt) mice characterized by elevated SAME levels spontaneously developed liver fibrosis at 3 months of age and HCC at 8 months of age (117). Zhang et al. (118) developed the Liver-specific O-linked β-N-acetylglucosamine (O-GlcNAc) transferase-KO (OGT-LKO) model, in which OGT-LKO mice exhibit hepatomegaly and ballooning degeneration at an early stage and progress to hepatic fibrosis and portal inflammation at 10 weeks of age, which can potentially be used as a novel, effective mouse model of liver fibrosis with broad translational implications for the screening and evaluation of anti-fibrotic drugs. Mdr2^−/−^ is also a widely used mouse model for the study of cholestatic liver fibrosis and cirrhosis. Deficiency of Mdr2 (a tubular phospholipid flipping enzyme) disrupts the secretion of biliary phospholipids, leading to increased bile secretion. Potentially toxic bile acids, which induce hepatocellular damage and cholestasis, are characterized by peribiliary inflammation and onion skin-type periductal fibrosis, similar to the pathology of primary sclerosing cholangitis (119). However, such transgenic/knockout mice can determine the role of the gene in liver fibrosis, but they are long in development, expensive and less used.

## 6. Immune induction methods

Autoimmune hepatitis can induce immune cells to attack their own hepatocytes under the influence of immunity or viral infection and other factors, resulting in inflammatory necrosis of the liver, followed by the development of liver fibrosis and cirrhosis. The model preparation method mainly includes schistosomiasis, virus, pig serum (PS), concanavalin A.

### 6.1. Schistosoma-induced liver fibrosis model

The main species of schistosomiasis that infect humans include *Schistosoma mansoni*, *Schistosoma haematobium* and *Schistosoma japonicum* (120)^.^ Infection by *S. japonicum* is a routine model for mechanistic or drug research purposes in liver fibrosis-related studies (121), and after infection, liver fibrosis is the main pathological manifestation of the disease. Schistosomiasis is a serious parasitic infection caused by the *S. haematobium*. Liver fibrosis in schistosomiasis occurs in the development of a complex series of hepatology involving immune inflammation, granuloma formation and liver injury (122). During schistosomiasis, where parasites deposit eggs in the host liver, inflammatory granulomas initially form around schistosomiasis eggs, and granulomatous reactions appear during the egg-laying period approximately 5–6 weeks after infection. As the granuloma matures, fibroblasts that lead to the production of extracellular matrix and collagen fibers are recruited in the outer zone of the granuloma, and dormant HSC are activated by various cytokines and transformed into myofibroblasts, leading to fibrosis (122–125).

Some studies selected mice percutaneously infected with cercariae of *S. japonicum* to establish a liver fibrosis model (126, 127). The results of the study (127) showed that compared with uninfected mice, mice infected with *S. japonicum* developed severe granulomatous inflammation and tissue fibrosis in the liver, spleen and large intestine 8 weeks after infection, the number of eosinophils was significantly increased by immunohistochemical staining with hematoxylin and eosin staining and CD68 macrophage-positive areas. CD4 helper cells, including Th1, Th2, Th17 and Treg cells, are also known to be involved in schistosomiasis egg-induced liver granulomatous inflammation and fibrosis. Lei et al. (128) found in mice that CD1d expression on hepatocytes was significantly reduced after infection with *S. japonicum*, accompanied by an increase in NKT cells, and an upregulation of Th1 and Th2 responses. During schistosomiasis infection, the eggs were trapped in the host liver and egg-derived products induce a polarized Th2 cell response leading to granuloma formation and eventual fibrosis (129). The proportion of γδ T cells producing and secreting IL-17A was significantly increased in the livers of mice infected with *S. japonicum*. In this mouse model of schistosomiasis infection, γδ T cells may promote liver fibrosis by recruiting CD11bGr-1 cells (130). In these models, the inducing mechanism of injury and the nature of the response, even if it leads to fibrosis, are of specific inflammatory and immune types, and the results may not be replicated in other fibrosis models. However, they highlighted the importance of the immune component in liver fibrosis (131).

### 6.2. Virus-induced liver fibrosis model

Human hepatitis B virus (HBV) belongs to the family hepatoviridae and is a small, enveloped, partially double-stranded DNA virus. Chronic HBV infection remains a major cause of liver injury and fibrosis. Individuals chronically infected with HBV can develop a range of liver diseases, ranging from liver fibrosis to cirrhosis to HCC. HBV infection leads to inflammatory changes followed by the release of different cytokines and chemokines such as IL-1 and IL-8, INF-γ and TNF-α. These cytokines and chemokines kill HBV-associated CD8+ cytotoxic T cells, this type of hepatic oxidative stress leads to activation of Kupffer cells, and then activation of HSC leads to fibrosis by triggering different genes (132, 133). Hepatitis C virus (HCV)-induced liver fibrosis mechanism is also one of the main causes of liver fibrosis. Hepatocyte specificity (CREBH) was identified as a key positive regulator of TGF-β2 transcription in HCV-infected cells. TGF-β2 released from infected cells may promote the cross-induction of TGF-β in an autocrine manner through its own signaling pathway, leading to increased fibrotic responses in adjacent HSCs (134).

Ye et al. (135) developed a mouse model of chronic HBV infection using adeno-associated virus serotype 8 (AAV8)-mediated delivery of the 1.2 kb HBV genome, which induces persistent HBV infection with hepatic fibrosis in immunocompetent mice; no animal model currently exists to mimic hepatic fibrosis during long-term HBV infection in immunocompetent mice. Therefore, this model can be used as a model to study the exact mechanism of liver fibrosis after chronic HBV infection and the potential development of new therapies. To closely mimic chronic hepatitis, Li et al. (136) used a replication-deficient recombinant adenoviral vector to deliver recombinant covalently closed circular DNA (rcccDNA) of HBV with site-specific DNA recombination to the liver and found a persistent necroinflammatory response and fibrosis in the mouse liver, with dysplastic lesions usually visible in the late stages of viral persistence, resembling the progressive pathology of clinical chronic hepatitis. HBV transgenic mice provide a reliable HBV replication model for studying the molecular mechanism of liver disease. However, viral genomes integrated into the host genome and in the immune system identify the virus as itself. The HBV genome cannot be eliminated from mouse hepatocytes because its use is limited to research purposes, antiviral drug screening and evaluation (137).

### 6.3. PS-induced liver fibrosis model

Serum as a heterologous antigen stimulates the immune response in experimental animals and stimulates the body to actively release cytokines to activate HSC, causing excessive deposition of ECM leading to liver fibrosis (138). The injection of porcine serum (PS) into model animals stimulates the production of antibodies to form immune complexes (IC) to activate complement, and the IC formed by long-term antigenic stimulation is deposited in the vascular wall, causing metaplasia resulting in vasculitis and perivasculitis, leading to liver injury and the formation of extensive progressive chronic inflammation, so that repeated hepatocyte degeneration, necrosis and hyperplasia gradually develop into fibrosis-like changes (139). Rats were injected intraperitoneally with 0.5 ml of PS twice a week for 16–24 weeks to induce liver fibrosis (138, 140). The PS-induced liver fibrosis model in rats exhibited changes similar to those of human liver disease (141). However, the modeling time is long and the experimental animals are prone to death due to allergic reactions.

### 6.4. Concanavalin A

Concanavalin A, a phytoagglutinin from knife-beans, is a common inducer of immune-mediated liver injury. The mechanism of liver fibrosis induction by knife-bezoar protein is to stimulate T-cell mitosis, promote the release of cytokines (TGF-β, TNF-α, etc.), cause an inflammatory response, and further the development of hepatitis into liver fibrosis (142). Immune-related liver fibrosis can be detected in mice after intravenous injection of Concanavalin A (143, 144). Some studies were performed by injecting Concanavalin A (10 mg/kg/wk./i.v) for 4–8 weeks in mice to induce hepatic fibrosis (145, 146).

Concanavalin A is a T cell-dependent model that causes immune-mediated hepatitis in a pattern similar to that induced by viral infection and is an ideal tool to study T cell-dependent immune-mediated liver injury (147, 148). Concanavalin A-induced liver fibrosis mimics that caused by autoimmune hepatitis, acute viral hepatitis or drug-induced immune activation in human immune-mediated liver fibrosis (149).

## 7. Combine induction methods

Some researchers can combine various factors to create an ideal animal model with more stable and precise mechanisms according to their model needs, and this combination of multiple factor approaches to create an animal model of liver fibrosis is called a composite model (63). Currently, the more widely used compound models are chemical and chemical, chemical and dietary, and transgenic and dietary combined induction.

### 7.1. Chemical + chemical

**Figure 2:**
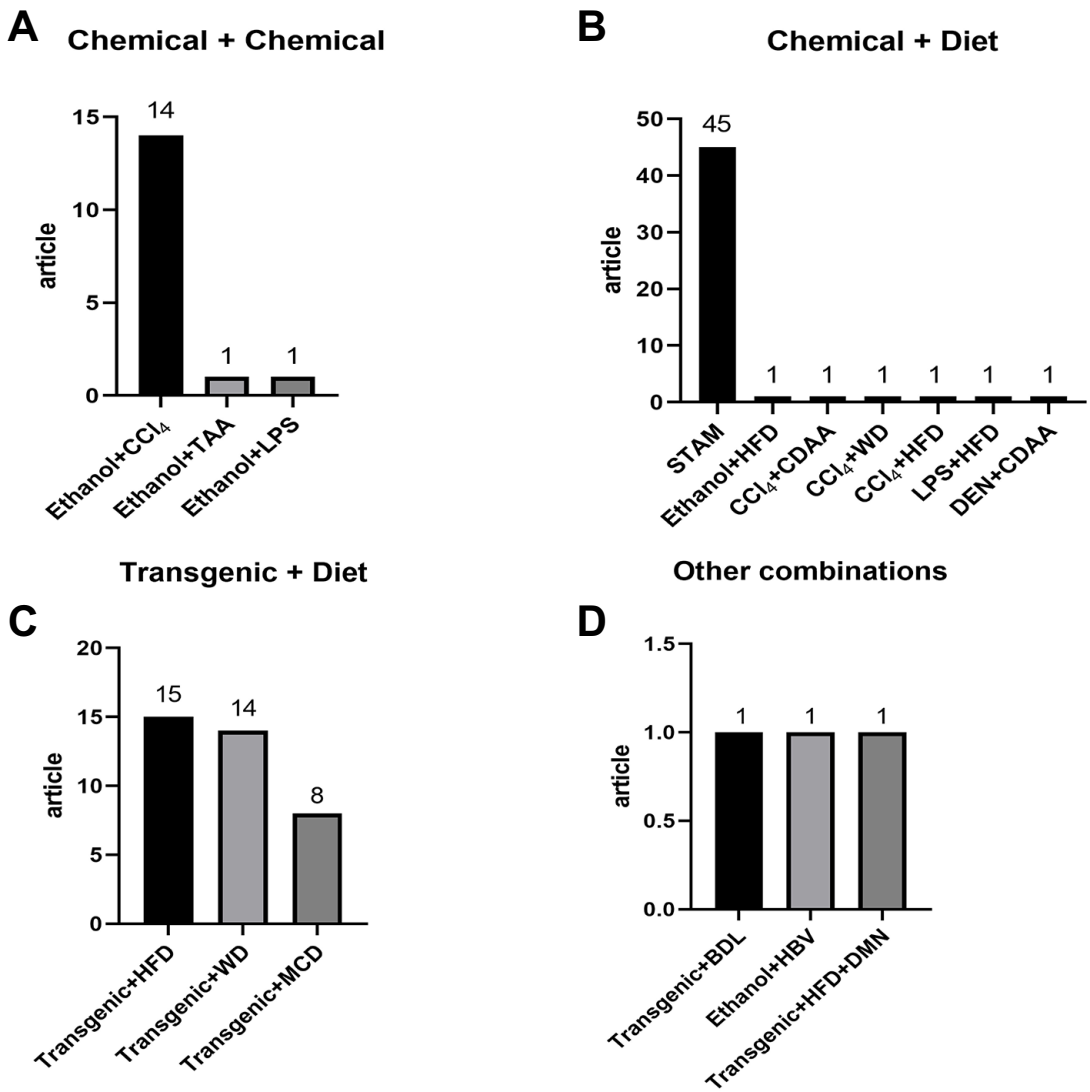
Detailed distribution of combine induction methods: **(A)** chemical + chemical: ethanol + CCl4 14, ethanol + TAA 1, ethanol + LPS 1; **(B)** chemical + diet: STAM 45, ethanol + HFD 1, CCl4 + CDAA 1, CCl4 + WD 1, CCl4 + HFD 1, LPS + HFD 1, DEN + CDAA 1; **(C)** transgenic + diet: transgenic + HFD 15, transgenic + WD 14, transgenic + MCD 8; **(D)** other combinations: transgenic + BDL 1, ethanol + HBV, transgenic + HFD + DMN 1.

Although the alcohol *ad libitum* feeding model can be used as a “stand-alone” model for mild alcoholic liver injury, more and more studies are combining it with other stressors to stimulate inflammation, fibrosis or HCC in the liver, and combined CCl_4_ and ethanol modeling is the most used model for chemical and chemically induced liver fibrosis (Figure 2). Brol et al. (150) treated mice with CCl_4_ plus ethanol (16%) for 7 weeks to induce mice that exhibited strong inflammation with significant liver fibrosis and moderate steatosis, a pattern mostly similar to the relationship between fibrosis, proliferation and inflammation in human alcoholic liver disease, providing a model for further basic research and drug trials. Some researchers induced liver fibrosis by administering ethanol and CCl_4_ together for 5–8 weeks, and liver sections showed typical pathological features, including marked steatosis, portal inflammation and necrosis, marked collagen deposition, hepatocellular fibrosis, and hepatocyte sparing (151, 152). Some researchers have also administered CCl_4_ intraperitoneally twice a week for the first 6 weeks, and then administered ethanol continuously to mice through a gastric feeding tube for 3 weeks, and saw a significant increase in liver injury, showing a clear “chicken wire” pattern of hepatic steatosis or steatohepatitis and liver fibrosis (153). It is evident that a reasonable dosing schedule, whether given simultaneously or at different times, can induce liver fibrosis.

### 7.2. Chemical + diet

To increase the severity of liver injury in a rodent NASH model, streptozotocin (STZ) (154), ethanol (155), CCl4 (8), and TAA (150) have been added to a modified diet. The STAM model is a model in which STZ is combined with HFD to induce liver fibrosis by administering a low dose of STZ to two-day-old neonatal C57BL/6 male mice given low doses of STZ and subsequently fed an HFC diet starting at 4 weeks of age. Mice developed hepatic steatosis and diabetes mellitus, reached steatohepatitis within 3 weeks, followed by cirrhosis within 8 weeks (i.e., approximately 12 weeks of age) and hepatocellular carcinoma within 16 weeks (154). Zhou et al. (155) developed a HFD plus binge drinking ethanol challenge model that mimics binge drinking and obesity in humans. Its data showed that alcohol abuse and HFD synergistically induced steatohepatitis and fibrosis (155, 156). HFD plus ethanol binge drinking characterized by neutrophilic liver infiltration resulted in significant upregulation of a range of genes associated with HSC activation and fibrogenesis compared to HFD feeding only. Current data from an HFD plus binge ethanol-fed mouse model suggest that obesity and binge eating act synergistically to promote liver fibrosis, which is mediated in part through the interaction of neutrophils and HSC (155).

Tsuchida et al. (8) established a mouse model of NASH by weekly use of high-fat, high-fructose and high-cholesterol WD combined with low-dose intraperitoneal injection of CCl_4_, which exhibited advanced fibrosis and rapid progression of HCC and mimicked the histological, immunological and transcriptomic features of human NASH. Related studies have shown that treatment of a mouse model of NASH with a combination of CCl_4_ and WD for more than 12 weeks induced the most severe steatosis as well as significant liver fibrosis and moderate inflammation (150), demonstrating the histological and transcriptomic profile of human NASH (8). Co-administration of TAA with a FFD to C57BL/6 J mice for 8 weeks, a novel model that exhibited liver inflammation and fibrosis in just 8 weeks, could be used for rapid screening of novel anti-NAFLD and hepatic anti-fibrotic agents (157). As with chemical methods combined with chemical methods of modeling, simultaneous administration, or separate administration, can induce different degrees of liver fibrosis. It is necessary to screen the appropriate liver fibrosis model according to the purpose of one’s study.

### 7.3. Transgenic + diet

The *ob* gene transcribes leptin, an adipocyte hormone involved in the regulation of food intake and insulin sensitivity. Functional leptin production is defective in *Lep^ob^/Lep^ob^* (*ob/ob*) mice (68). The *ob/ob* mice are well known models of extreme obesity and insulin resistance (158). The *Lepr^db^*/*Lepr^db^* (*db/db*) model has a metabolic phenotype similar to that of ob/ ob animals and exhibits leptin resistance caused by premature termination of leptin receptor transcription, a similar mutation exists in rats and has been described as *Lepr^fa^/Lepr^fa^* (*fa/fa*), the *fa/fa* model exhibits a phenotype similar to that of *ob/ ob* and *db/db* mice with spontaneous onset of severe obesity, insulin resistance and steatosis (22, 68). However, liver inflammation and fibrosis in genetically defective *ob/ob*, *db/db* mice, *fa/ fa* rats, or partially transgenic mice models are mild and can induce varying degrees of inflammation and liver fibrosis when combined with dietary measures (feeding MCD or HFD diet) (159). Kim et al. (160) fed *ob/ob* mice to a HFD for 20 weeks to establish an animal model of NASH with fibrosis. Treatment of *ob/ob* mice fed a long-term high-fat diet resulted in significant weight loss, adipose tissue hypertrophy and inflammation, hepatic steatosis, inflammation and fibrosis, and insulin resistance >1 year (161). MCD diet induces hepatic inflammation and fibrosis in *PPARα^−/−^* mice (162). This mouse model has been widely used to cause severe steatohepatitis and fibrosis, similar to human non-alcoholic steatohepatitis pathology (163). The pathogenesis involves the hepatic oxidative stress observed in human NASH (164). Chen et al. (165) gave adropin-deficient (adropin-KO) mice fed MCD diet for 4 weeks or WD diet for 16 weeks, adropin-KO mice exhibited more severe hepatic macrosteatosis, inflammation and ballooning with significantly higher NASA scores and increased areas of fibrosis with marked perisinusoidal fibrosis; fibrosis-related genes such as Col1a1, Acta2 and inflammation-related genes such as IL1b, IL6 and TNF were also induced in large numbers in Adropin-KO livers.

Most of the animal models of compound liver fibrosis use 2 methods in combination, and 3 methods are used in combination (166), but rarely (Figure 2). With the development of society and the continuous improvement of living standards, modern human life has a certain complexity, which leads to the complex and variable factors of liver disease, and even a variety of compound factors together affect the formation and development of liver disease (167). To some extent, the compound model solves the problem that there is a gap between the single-factor animal model and the modern clinical patient’s condition, and the compound animal model of liver fibrosis has a high modeling rate and a low morbidity and mortality rate of animals during the modeling period.

## 8. Discussions and prospect

It is now widely accepted that liver fibrosis is a reversible process and that early treatment can inhibit the progression of fibrosis or even reverse it, thus attracting a large number of researchers to study the therapeutic field of liver fibrosis. There are a hundred different treatment areas for liver fibrosis, including general therapy (exercise, dietary interventions), drug therapy, herbal therapy, stem cell therapy, gene therapy, natural substance therapy, biomaterial therapy, surgical therapy, molecular level therapy, microbial therapy, combination therapy, etc. Most of these fundamental articles for the treatment of liver fibrosis use animal experimental models for validation. Therefore, we conducted a PubMed search using the search term “liver fibrosis” “treatment” between 2017 and 2022 to collect articles on basic research in the field of liver fibrosis treatment, and a total of 2,518 articles used animal models of liver fibrosis.

**Figure 3:**
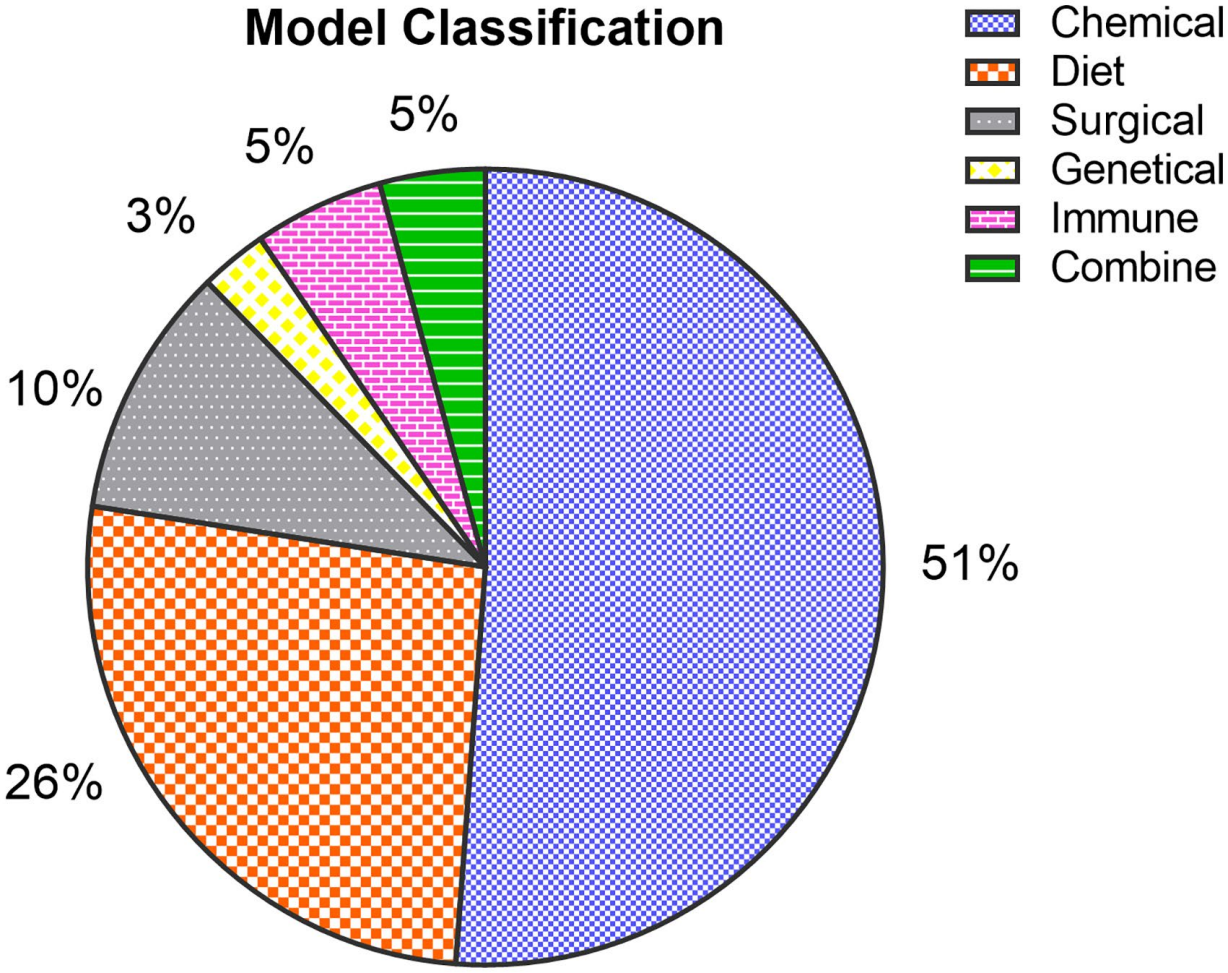
Classification of fibrosis models: Among the collected articles, 1,289 (51%) were chemically induced models, 661 (26%) were diet-induced models, 258 (10%) were surgically induced models, 69 (3%) were transgene-induced models, 133 (5%) were immune-induced models, and 108 (5%) were combine-induced animal models.

**Figure 4:**
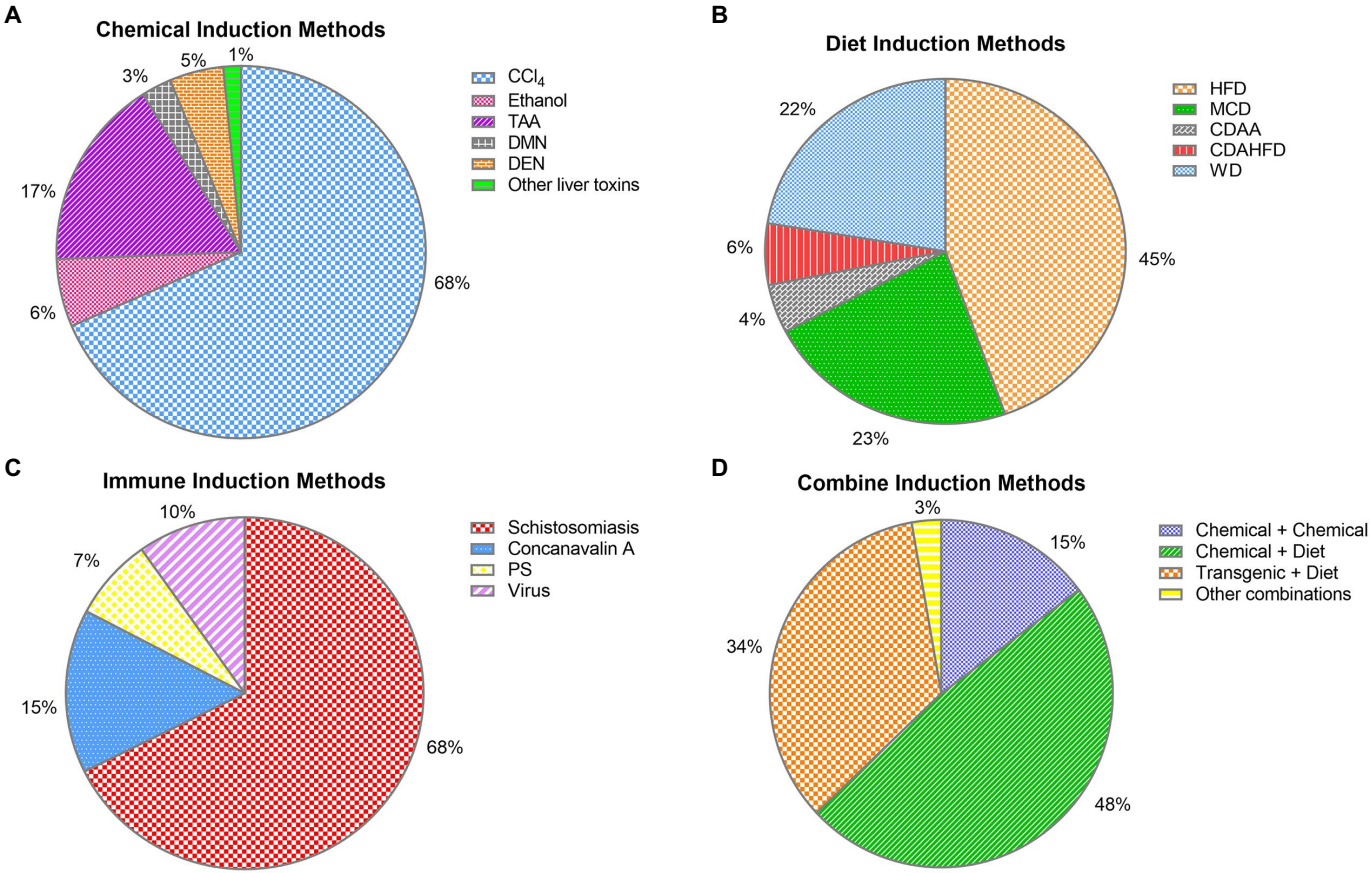
Specific distribution of liver fibrosis models: **(A)** chemical-induced liver fibrosis models were specifically distributed: CCl_4_ 883 (68%), TAA 215 (17%), ethanol 76 (6%), DEN 61 (5%), DMN 34 (3%), other liver toxins 20 (1%); **(B)** diet-induced liver fibrosis models were specifically distributed: HFD 295 (45%), WD 148 (22%), MCD 150 (23%), CDAHFD 38 (6%), CDAA 30 (4%); **(C)** immune-induced liver fibrosis models were specifically distributed: Schistosomiasis 90 (68%), concanavalin A 20 (15%), virus 13 (10%), and porcine serum 10 (7%). **(D)** combined-induced liver fibrosis was specifically distributed: chemical + chemical 16 (15%), chemistry + diet 52 (48%), transgenic + diet 37 (34%), and other combined methods 3 (3%).

**Figure 5:**
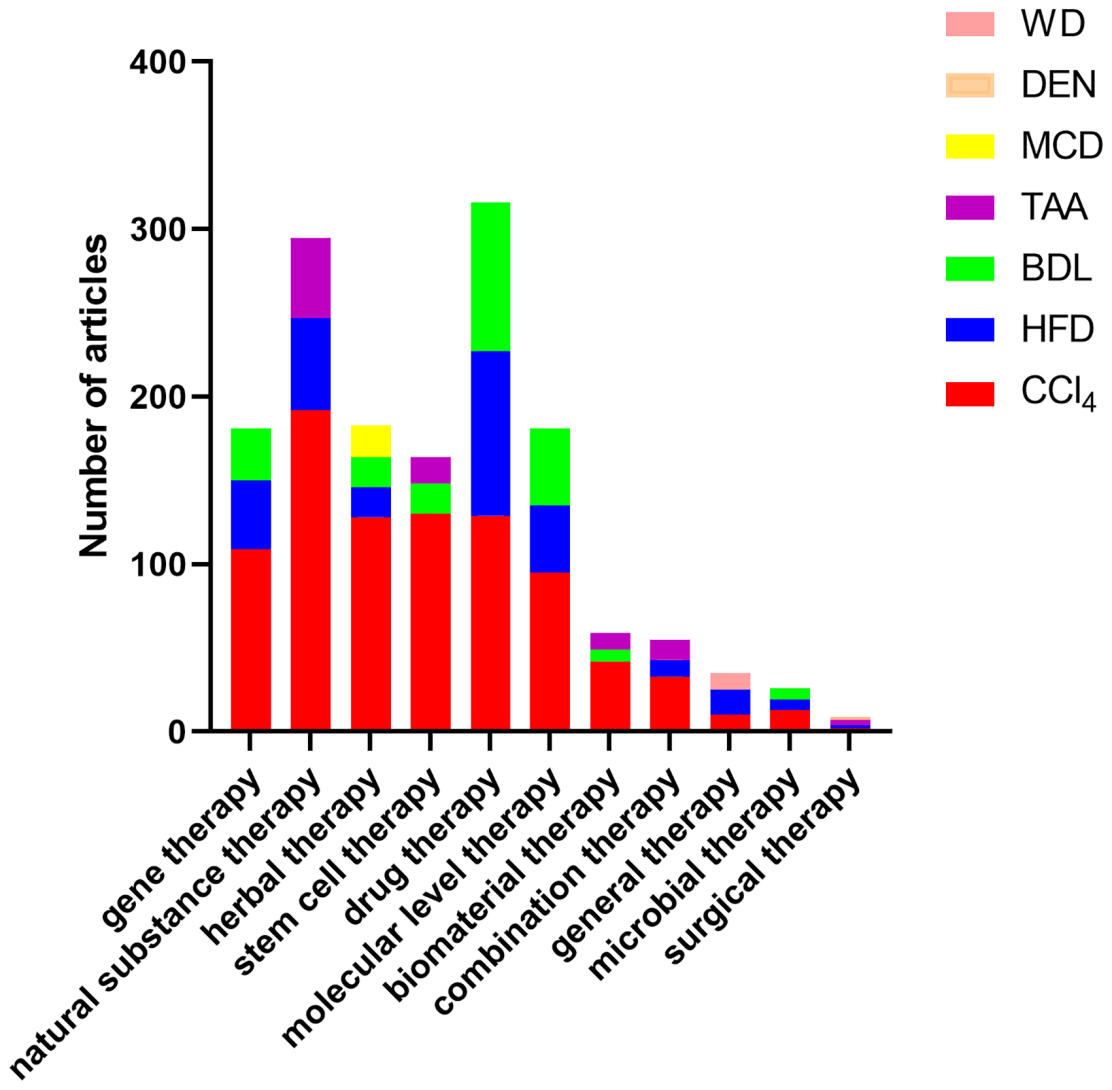
Top three animal models used in articles in the field of liver fibrosis treatment: Among the collected articles, the details of the top three animal models used in articles in each field of treatment are as follows: gene therapy: CCl_4_ (109), HFD (41), BDL (31); natural substance therapy: CCl_4_ (192), HFD (55), TAA (48); herbal therapy. CCl_4_ (128), MCD (19), BDL (18), HFD (18); stem cell therapy: CCl_4_ (130), BDL (18), TAA (16); drug therapy: CCl_4_ (129), HFD (98), BDL (89); biomaterial therapy: CCl_4_ (42), TAA (10), BDL (7); microbiological treatment: CCl_4_ (13), BDL (7), HFD (6); surgical treatment: TAA (3), DEN (2), CCl_4_ (2), HFD (2); general treatment: HFD (15), WD (10), CCl_4_ (10); molecular level treatment: CCl_4_ (95), BDL (46), HFD (40); combination therapy: CCl_4_ (33), TAA (12), HFD (10).

The results from the collected data show that transgenic-induced liver fibrosis models are relatively less used, which may be attributed to the long development time and high price of this model (Figure 3). Chemical injury-induced liver fibrosis models are the most widely used (Figure 3), and these models use chemical drugs to enter hepatocytes to produce toxic metabolites that cause persistent liver injury and induce the formation of liver fibrosis. Among them, the CCl4-induced liver fibrosis animal model is similar to human liver fibrosis in some aspects of morphology and pathophysiology, and is the most used animal modeling method for liver fibrosis because of its short modeling time, low cost, and high reproducibility (Figures 4, 5). Animals in the CCl4-induced liver fibrosis model do not become obese or develop insulin resistance, which is very different from the pathophysiological features of patients with non-alcoholic fatty liver disease induced by metabolic disorders. The most common signs of fibrosis in NASH are mainly caused by excessive consumption of high-fat components, where patients absorb nutrients The HFD-induced liver fibrosis model overcomes the shortcomings of the MCD-induced liver fibrosis model, in which animals with increased body weight and peripheral insulin resistance develop and mimic the etiology of the disease by replicating poor dietary habits, with phenotypic features similar to those of human nonalcoholic steatohepatitis. Fibrosis model, with a short modeling period, simple method and no need for exposure to toxic substances, is currently a common method for inducing cholestatic liver fibrosis models to study diseases related to biliary obstruction. As shown by our collected data, CCl4, HFD and BDL-induced liver fibrosis models, relative to other methods, are widely used in the basic field of liver fibrosis treatment (Figures 4, 5).

So far, researchers have successfully developed many models of liver fibrosis using different experimental animals and different methods. Each model has its disadvantages and advantages, and a reasonable method of model preparation needs to be selected according to the experimental purpose and requirements.

## Author contributions

SW collected the data and wrote the manuscript. XW, WX, FL, ML, and KL revised the manuscript. YH and JW provided constructive comments on the review. All authors contributed to the article and approved the submitted version.

## Funding

We acknowledge financial support from the Wuhan University of Science and Technology Graduate School Scholarship (No. ZY24001), the Hubei Provincial Health and Health Commission Research Project (No. WJ2023M121), and the WUST startup fund (Chu Tian Scholars Program). Figure 1 was created with BioRender.com.

## Conflict of interest

The authors declare that the research was conducted in the absence of any commercial or financial relationships that could be construed as a potential conflict of interest.

## Publisher’s note

All claims expressed in this article are solely those of the authors and do not necessarily represent those of their affiliated organizations, or those of the publisher, the editors and the reviewers. Any product that may be evaluated in this article, or claim that may be made by its manufacturer, is not guaranteed or endorsed by the publisher.
